# Effective Protection Induced by a Monovalent DNA Vaccine against Dengue Virus (DV) Serotype 1 and a Bivalent DNA Vaccine against DV1 and DV2 in Mice

**DOI:** 10.3389/fcimb.2017.00175

**Published:** 2017-05-12

**Authors:** Xiaoyan Zheng, Hui Chen, Ran Wang, Dongying Fan, Kaihao Feng, Na Gao, Jing An

**Affiliations:** ^1^Department of Microbiology and Parasitology, School of Basic Medical Sciences, Capital Medical UniversityBeijing, China; ^2^Beijing Tropical Medicine Research Institute, Beijing Friendship Hospital, Capital Medical UniversityBeijing, China; ^3^Center of Epilepsy, Beijing Institute for Brain DisordersBeijing, China

**Keywords:** dengue virus, DNA vaccine, monovalent vaccine, bivalent vaccine, electroporation, dengue

## Abstract

Dengue virus (DV) is the causal pathogen of dengue fever, which is one of the most rapidly spread mosquito-borne disease worldwide and has become a severe public health problem. Currently, there is no specific treatment for dengue; thus, a vaccine would be an effective countermeasure to reduce the morbidity and mortality. Although, the chimeric Yellow fever dengue tetravalent vaccine has been approved in some countries, it is still necessary to develop safer, more effective, and less costly vaccines. In this study, a DNA vaccine candidate pVAX1-D1ME expressing the prME protein of DV1 was inoculated in BALB/c mice via intramuscular injection or electroporation, and the immunogenicity and protection were evaluated. Compared with traditional intramuscular injection, administration with 50 μg pVAX1-D1ME via electroporation with three immunizations induced persistent humoral and cellular immune responses and effectively protected mice against lethal DV1 challenge. In addition, immunization with a bivalent vaccine consisting of pVAX1-D1ME and pVAX1-D2ME via electroporation generated a balanced IgG response and neutralizing antibodies against DV1 and DV2 and could protect mice from lethal challenge with DV1 and DV2. This study sheds new light on developing a dengue tetravalent DNA vaccine.

## Introduction

Dengue virus (DV) is a member of the family *Flaviviridae* and contains four distinct serotypes (DV1-4). DV infections cause either asymptomatic disease or some clinical illnesses ranging from self-limited dengue fever (DF) to severe dengue (sDF), including dengue hemorrhagic fever and dengue shock syndrome (Bhatt et al., 2013); dengue is the most important arbovirus disease in the world in terms of the highest morbidity and mortality (Porter and Raviprakash, 2015). It was reported that there were 58.4 million symptomatic DV infections with 13,586 fatal cases in 2013, and the global cost is 8.9 billion US dollars annually (Shepard et al., 2016). As a major public health problem, dengue is considered to be one of the fastest growing epidemics by the World Health Organization (Arima et al., 2015; Rogers, 2015). In its global strategy for dengue control, the World Health Organization aims to reduce dengue mortality and morbidity by at least 50 and 25%, respectively, by 2020 (WHO, 2012).

Since the first outbreak in Guangdong province in 1978, dengue has broken out several times in the Hainan, Fujian, Guangxi, and Zhejiang provinces in mainland China in recent years (Wu et al., 2010; Lin et al., 2016). In these dengue outbreaks, all four dengue serotypes were found to be co-circulating in endemic areas, but DV1 is the predominant serotype. In 2014, the Guangdong province of China suffered from the most serious dengue outbreak in its history, and the total number of DF cases was more than 45,000 (Huang et al., 2016). In the outbreak, co-circulation of DV1 and DV2 was identified, and some isolates of DV1 or DV2 were closely related with Guangzhou isolates from previous years; the frequency of DV1 epidemics was still higher than that of DV2 (Zhang et al., 2014; Ren et al., 2015), indicating that dengue became endemic in Guangdong and is no longer an imported disease in China (Lin et al., 2016; Zhao et al., 2016). Therefore, controlling dengue is a long-term effort, and developing a vaccine is believed to be the most reliable approach to achieve this goal (Hermann et al., 2015).

Theoretically, a secondary DV infection of heterotypic serotype may increase the risk of sDF in patients and it is the major barrier for developing successful vaccine against DVs. The reason is not very clear currently, but the more accepted interpretation is the role of antibody dependent enhancement (ADE) (Cummings et al., 2005). Therefore, optimal dengue vaccines should induce a balanced immune response to all four DV serotypes. A DNA vaccine, as a simple and efficient technique with attractive advantages including inexpensiveness, ease of production, stability for storage and shipping, may overcome the obstacle of ADE through balanced and long-term expression of immunogens of all four DV serotypes.

The DV genome contains a single open reading frame and encodes three structural proteins: the capsid protein (C), the precursor of membrane protein (prM), and the envelope protein (E), followed by seven non-structural proteins. Among the structural proteins, the prM and E proteins are major target molecules for developing vaccines because the E protein contains the immunological epitopes for inducing humoral and cellular immune responses, and the prM protein is essential for the correct conformation of the E protein during the viral maturation (Bray and Lai, 1991). Therefore, the *prM* and *E* genes are the principal molecular candidates for developing flavivirus DNA vaccines.

In our previous studies, DNA vaccine candidates expressing the prM and E proteins of DV1 or DV2 with the eukaryotic expression vector pCAGGSP7 have been demonstrated to induce some immune protection at three doses (100 μg each) of DNA delivered by intramuscular (IM) injection (Zheng et al., 2011; Lu et al., 2013). Recently, to translate the DNA vaccine candidates for further clinical application, the plasmids were reconstructed using pVAX1, a unique US FDA-approved vector for developing DNA vaccines; it was confirmed that the DNA vaccine candidate pVAX1-D2ME containing the *prM* and *E* genes of DV2 could protect mice from lethal DV2 infection and induce an effective specific antibody response in rabbits (Chen et al., 2016). These findings establish an important foundation for further researching a DV1 vaccine.

In this study, another DNA vaccine candidate, pVAX1-D1ME, encoding the prM and E proteins of DV1 was constructed and vaccinated in mice via IM injection or *in vivo* electroporation (EP); immune responses and protection were determined. Based on this, the immunogenicity and immuno-protection of a bivalent vaccine candidate consisting of pVAX1-D1ME and pVAX1-D2ME was evaluated in mice to explore the possibility of a bivalent vaccine against DV1 and DV2 and simultaneously lay the foundation for the study of a tetravalent DNA vaccine against all DV serotypes. Our results showed that the immunization of pVAX1-D1ME via EP could induce persistent humoral and cellular immune responses and effectively protect mice against lethal DV1 challenge. The immunization of a bivalent vaccine consisting of pVAX1-D1ME and pVAX1-D2ME via EP could generate a balanced IgG response against DV1 and DV2 and protect mice from lethal challenge with DV1 and DV2. Moreover, no increased clinical signs were observed in the mice. This study encourages further development of a dengue tetravalent DNA vaccine.

## Materials and methods

### Animals and ethics statement

Six-week-old female BALB/c mice were purchased from Vital River Laboratories (Beijing, China) and were maintained in specific pathogen-free environments. Animal care and experimental procedures were approved by the Institutional Animal Care and Use Committee of Chinese Capital Medical University (approval number: AEEI-2015-066).

### Cells and viruses

Vero cells were grown at 37°C in minimal essential medium (MEM) supplemented with 5% fetal bovine serum (FBS). Baby hamster kidney (BHK) cells were grown at 37°C in MEM supplemented with 10% FBS. *Aedes albopictus* C6/36 cells were grown at 28°C in RPMI 1640 supplemented with 10% FBS. Mice fibroblast L929 cells were grown in RPMI 1640 supplemented with 10% FBS at 37°C.

The DV1 (Hawaii strain) and the DV2 (New Guinea C strain) used in challenge model were clinical isolates provided by Guangdong Center for Disease Control and Prevention. Viruses were propagated in C6/36 cells and their titers were determined by plaque assays on Vero cells.

### Construction of plasmids

The DNA vaccine candidate was constructed for the expression of DV1 prME using the eukaryotic expression vector pVAX1 (Invitrogen, USA). Briefly, the fragments of DV1 *prM* and *E* (GenBank accession number U88535.1, nucleotides 365 to 2419 bp) were amplified by polymerase chain reaction from a full-length infectious clone of DV1. The forward and reverse primers were as follows: 5′-GCGTTC**GCTAGC**ATGGCAATGTTGAACATAATGAAC-3′ and 5′-GGCACA**CTCGAG**TTACGCCTGAACCATGACTCCTAG-3′. The gene of interest was finally sub-cloned into pVAX1, and the recombinant plasmid was named as pVAX1-D1ME, which was further verified by enzyme digestion and DNA sequencing.

### Indirect immunofluorescence (IFA) staining

To detect the expression of the recombinant plasmid in eukaryotic cells, BHK cells (0.5 × 10^5^ per well) in a 24-well plate were transfected with the pVAX1-D1ME and served as the antigen. The transfection was performed with Lipofectamine 2000 (Invitrogen, USA) as specified by the supplier's instruction. Transfected BHK cells were fixed with 4% PFA, permeabilized with 0.2% Triton X-100 in PBS and then blocked with 1% bovine serum albumin in PBS. The DV1-infected mouse serum diluted 1:1,000 was used as the primary antibody. The secondary antibody was goat anti-mouse IgG conjugated with FITC (EarthOx, USA) diluted 1:500. DAPI (Sigma, USA) diluted 1:2500 was used to stain nucleus. After mounting with 40% glycerol, the cells were examined and photographed under a fluorescence microscope (Olympus BX61, Japan). Cells transfected with the vector pVAX1 served as the negative control.

### Vaccination of pVAX1-D1ME

To evaluate the immunogenicity of pVAX1-D1ME with different dosage and delivery methods, female BALB/c mice were divided into four groups (EP50, EP5, IM50, and pVAX1 groups, Table 1). In the IM50 group, each mouse was immunized with 50 μg pVAX1-D1ME into the quadriceps muscle of the hind limb by a syringe. In the EP groups, two silver needles 6 mm apart were inserted over the injection site, 5 μg or 50 μg pVAX1-D1ME was injected into the muscle, and then six electric pulses (36 V, 10 ms) were applied using the gene delivery device (Terasa Healthcare Sci-Tech, China). Mice inoculated with 50 μg pVAX1 via EP served as the negative controls. The mice were immunized three times at two-week intervals.

**Table 1:** **Mouse immunization groups and scheme**.

**Groups**	**Immunogen**	**Gene delivery**	**Dose (μg)**	**Vaccination times, interval (weeks)**	**EP Voltage (V)**	**EP Pulse length (ms)**
**EVALUATING THE IMMUNE RESPONSES OF pVAX1-D1ME (4 GROUPS)**						
EP50	pVAX1-D1ME	EP	50	3, 2	36	10
EP5	pVAX1-D1ME	EP	5	3, 2	36	10
IM50	pVAX1-D1ME	IM	50	3, 2	–	–
pVAX1	pVAX1	EP	50	3, 2	36	10
**DETECTING THE OPTIMAL IMMUNIZATION TIMES OF pVAX1-D1ME (4 GROUPS)**						
Once-EP50	pVAX1-D1ME	EP	50	1, 0	36	10
Twice-EP50	pVAX1-D1ME	EP	50	2, 2	36	10
Thrice-EP50	pVAX1-D1ME	EP	50	3, 2	36	10
pVAX1	pVAX1	EP	50	3, 2	36	10
**EVALUATING THE IMMUNE RESPONSES OF THE BIVALENT VACCINE (3 GROUPS)**						
B-EP	pVAX1-D1ME pVAX1-D2ME	EP	50^a^ 50^a^	3, 2	36	10
B-IM	pVAX1-D1ME pVAX1-D2ME	IM	50^b^ 50^b^	3, 2	–	–
B-pVAX1	pVAX1	EP	50^c^ 50^c^	3, 2	36	10

a *The mice were immunized with 50 μg pVAX1-D1ME and 50 μg pVAX1-D2ME via IM into limbs bilaterally, then six electric pulses (36V, 10 ms) were applied using the gene delivery device*.

b *The mice were immunized with 50 μg pVAX1-D1ME and 50 μg pVAX1-D2ME via IM into limbs bilaterally*.

c *The mice were immunized with 50 μg pVAX1 via IM into bilateral limbs, then six electric pulses (36V, 10 ms) were applied using the gene delivery device*.

To determine the appropriate immunization schedule, the mice were divided into four groups (once-EP50, twice-EP50, thrice-EP50, and pVAX1 groups, Table 1), and immunized with 50 μg pVAX1-D1ME via EP once, twice or three times at two-week intervals, respectively. Mice inoculated with 50 μg pVAX1 via EP three times served as the negative controls.

### Immunization of the bivalent vaccine consisting of pVAX1-D1ME and pVAX1-D2ME

To evaluate the immunogenicity of the bivalent vaccine consisting of pVAX1-D1ME and pVAX1-D2ME, BALB/c mice were divided into three groups (Table 1). In the B-IM group, mice were immunized with 50 μg pVAX1-D1ME and 50 μg pVAX1-D2ME into the quadriceps muscles of the left and right hind limbs via IM injection. In the B-EP group, mice were immunized via EP immediately after IM injection with 50 μg pVAX1-D1ME and 50 μg pVAX1-D2ME. In the B-pVAX1 group, mice were inoculated via EP immediately after IM injection with 100 μg pVAX1 (50 μg in each leg), and served as the negative controls. The immunization was performed three times with two-week intervals.

### Immunohistochemistry (IHC) staining

IHC staining was conducted to detect the expression of the DV1 prME protein in the local injection site. Two weeks after the third immunization, the thigh muscles of mice were isolated and cut into 5-μm consecutive paraffin sections. Following the manufacturer's instructions, the sections were processed for IHC analyses using the PV6001 IHC detection reagent (ZSGB-BIO, China). Briefly, the slides were blocked in 1% bovine serum albumin at 37°C for 1 h. The DV1-infected mouse serum diluted 1:500 was used as the primary antibody and incubated at 4°C overnight. The secondary antibody was HRP-conjugated goat anti-mouse IgG (SANTA, USA) diluted 1:2,000 at 37°C for 1 h.

### Enzyme-linked immunosorbent assay (ELISA)

Serum samples were collected by tail bleeding, and specific IgG antibodies against DV1 or DV2 were determined by ELISA. Briefly, each well of the 96-well microtiter plate was coated with 2 μg of the concentrated DV1 or DV2 protein from the infected C6/36 cells, and followed by blocking with 2% bovine serum albumin. The plate was incubated with two-fold serial dilutions of the serum samples (started from 1:100), then antibody titers were detected with HRP-conjugated goat anti-mouse IgG (1:5,000, SANTA, USA), and substrate solution of orthophenylene diamine. The absorbance at 492 nm was measured using a microplate reader (Thermo, USA). The highest dilution, yielding an optical density (OD) greater than that of the negative control at the same dilution, was recorded as the end-point titer.

To determine the IgG subclasses, two weeks after the third immunization, antibody isotype ELISA was performed using serum samples (1:100) as the first antibody, and goat anti-mouse IgG1-HRP or IgG2a-HRP (1:4,000, SouthernBiotech, USA) was used as the secondary antibody.

### Plaque reduction neutralization test (PRNT)

Two weeks after the third immunization, serum samples were collected to detect the levels of anti-DV1 neutralizing antibody (NAb) by PRNT. After heating at 56°C for 30 min to inactivate complement, sera were two-fold diluted (starting from 1:10) in MEM containing 2% FBS. Diluted sera were mixed 1:1 with DV1 suspension containing 100 plaque-forming units (PFU), and incubated at 37°C for 1 h. Then, the mixture was added to Vero cell in duplicate wells of a 24-well plate, and incubated at 37°C for another 1 h. The infected Vero cells were washed then layered with MEM containing 2% FBS and 1.1% methylcellulose. After incubation at 37°C for 8 days in 5% CO_2_, the plaques were stained with crystal violet and counted. The maximum serum dilution that yielded a 50% plaque reduction (PRNT_50_) compared to the average plaque number of the virus control wells was calculated as the NAb titer.

### Enzyme-linked immunospot (ELISPOT) assay

Two weeks after the third immunization, splenocytes were isolated aseptically from mice to measure the generation of interferon (IFN)-γ, interleukin (IL)-2, IL-4, and IL-10 using ELISPOT kits per the manufacturer's instructions (BD Biosciences, USA). Briefly, splenocytes (1 × 10^6^ cells/well) were added into 96-well plates (Millipore, USA) pre-coated with 100 μl (1.0 mg/ml) of capture antibodies, and stimulated with 5 μg/well of concentrated DV1 proteins or concanavalin A (0.5 μg/well, positive control) or RPMI 1640 medium (negative control) at 37°C for 60 h. The cells were then incubated with secondary biotinylated detection antibodies, streptavidin-HRP (BD Biosciences, USA), and substrate (AEC Chromogen). The spot-forming units (SFU), which represented the positive single cells, were counted automatically using an ELISPOT reader (CTL, USA).

### Cytotoxic T lymphocyte (CTL) activity

Two weeks after the third vaccination, freshly isolated mouse splenocytes were stimulated with concentrated DV1 proteins (2 μg/10^5^ cells) for 72 h to prepare effector cells. L929 cells infected with DV1 (multiplicity of infection = 10) for 16–18 h were used as target cells. In U-bottom 96-well plates, the target cell suspension (5 × 10^3^ cells/well) was dispensed with the effector cells at various effector: target (E:T) ratios of 100:1, 50:1, 25:1, and 5:1. After incubation for 5 h at 37°C and 5% CO_2_, 50 μl/well of the supernatant was transferred to 96-well flat-bottom plates. The lactate dehydrogenase (LDH) activity was determined by the CytoTox non-radioactive cytotoxicity assay kit (Promega, USA) per the manufacturer's instructions. The absorbance at 490 nm was recorded on a microplate reader, and the percentage of specific lysis (% LDH release) was calculated using the following formula: % cytotoxicity = 100 × (experimental release – effector spontaneous release – target spontaneous release)/(target maximum release – target spontaneous release).

### Lymphocyte proliferation ability

Two weeks after the third inoculation, 100 μl of freshly isolated mouse splenocytes (1 × 10^6^ cells) were stimulated with concentrated DV1 proteins (100 μg/ml) or ConA (5 μg/ml, positive control) at 37°C for 72 h. Then, 10 μl of the CCK-8 solution (Dojindo, Japan) was added to each well, and incubated for 5 h. The OD at 450 nm was detected using a microplate reader. The stimulation index (SI) was calculated as (OD_stimulated_ − OD_blank_) / (OD_unstimulated_ − OD_blank_).

### Protection test

Two weeks after the last immunization, the mice were challenged with 50 LD_50_ of DV1 (2 × 10^7^ PFU) or 30 LD_50_ of DV2 (600 PFU) intracerebrally (Chen et al., 2016). Body weight, clinical signs, and mortality were monitored daily for 30 days. Clinical symptoms were scored as follows: 0, healthy; 1, ruffled hair or hunchbacked appearance; 2, asthenia, wasting or bradykinesia; 3, forelimb or hindlimb weakness; 4, paralysis or moribundity; and 5, death.

### Statistical analysis

All data were analyzed using SPSS software (version 17.0). Kaplan-Meier survival curves and the log rank test were used for survival analysis. Weight changes and sign scores were analyzed by the repeated measures analysis of variance. Others were analyzed by one way analysis of variance. Differences were considered to be statistically significant and highly significant at *p* < 0.05 (^*^) and *p* < 0.01 (^**^), respectively.

## Results

### Expression of the prME protein in BHK cells and the injection site of immunized mice

**Figure 1:**
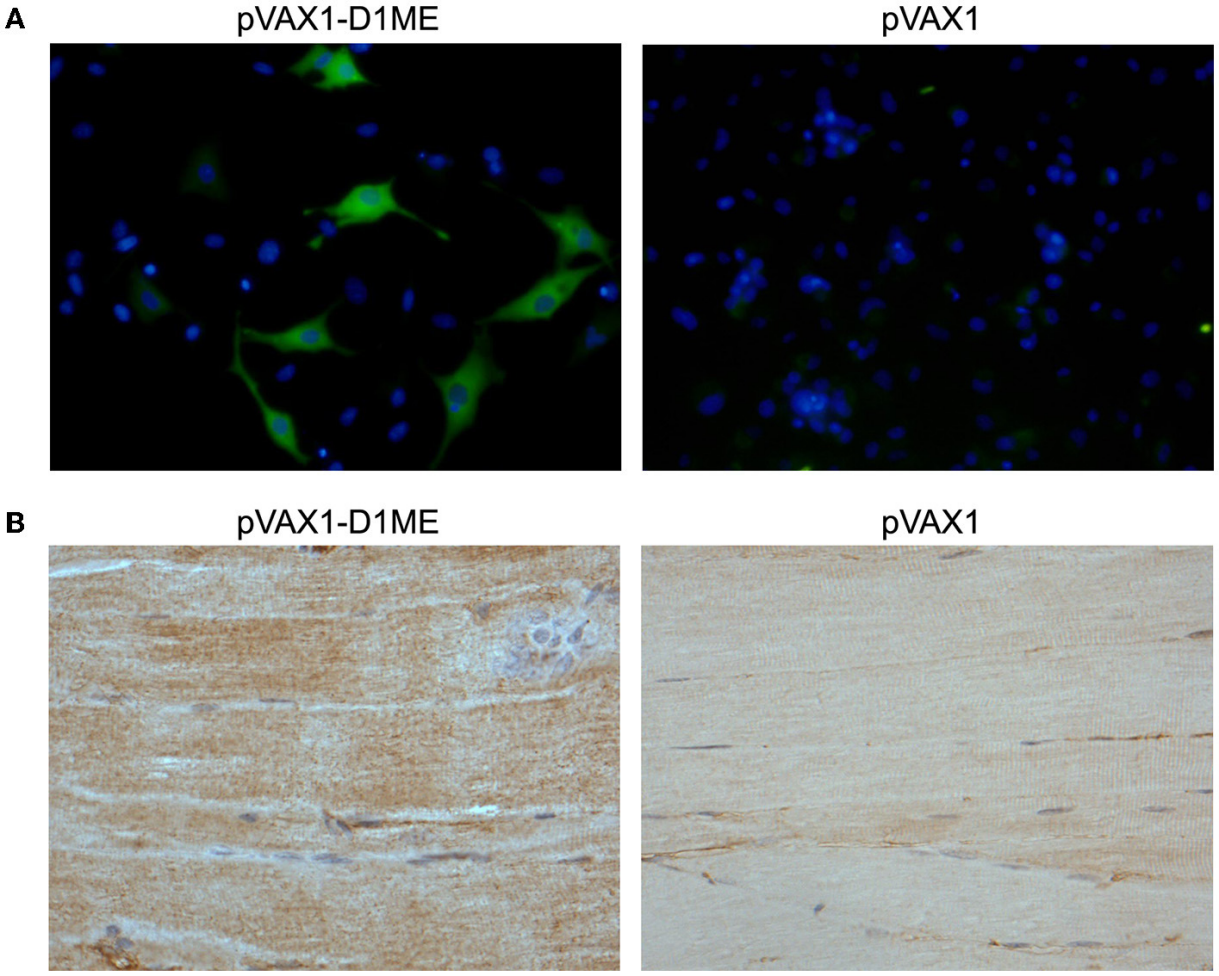
**Determination of prME protein expression. (A)** *In vitro* expression of the prME protein in DNA-transfected BHK cells by IFA (fluorescence microscopy, × 400). **(B)** *In vivo* expression of the prME protein in tissues by IHC. The muscle tissues were collected from mice sacrificed two weeks after the third immunization with 50 μg DNA (light microscopy, × 200).

To determine the *in vitro* expression of the prME protein in eukaryotic cells by IFA, the BHK cells were transfected with the plasmids pVAX1-D1ME and pVAX1. As shown in Figure 1A, the BHK cells that received the pVAX1-D1ME exhibited intense fluorescence in the cytoplasm; while negative control cells received the pVAX1 vector failed to show any specific fluorescence. This result confirmed that the pVAX1-D1ME was successfully transfected into eukaryotic cells and worked effectively. Thus, this plasmid could be used for further experiments.

Furthermore, to determine the *in vivo* expression of the prME protein by IHC, the muscle tissues were sampled from mice sacrificed two weeks after the third immunization. As shown in Figure 1B, expression of the protein of interest was detected in the muscle tissue of the mouse that received the pVAX1-D1ME but not the pVAX1 vector. This result confirmed that the pVAX1-D1ME was successfully transfected into muscle cells via EP and worked effectively. Thus, this plasmid could be used as the DNA vaccine candidate in further experiments.

### Antibody response to immunization with the pVAX1-D1ME

**Figure 2:**
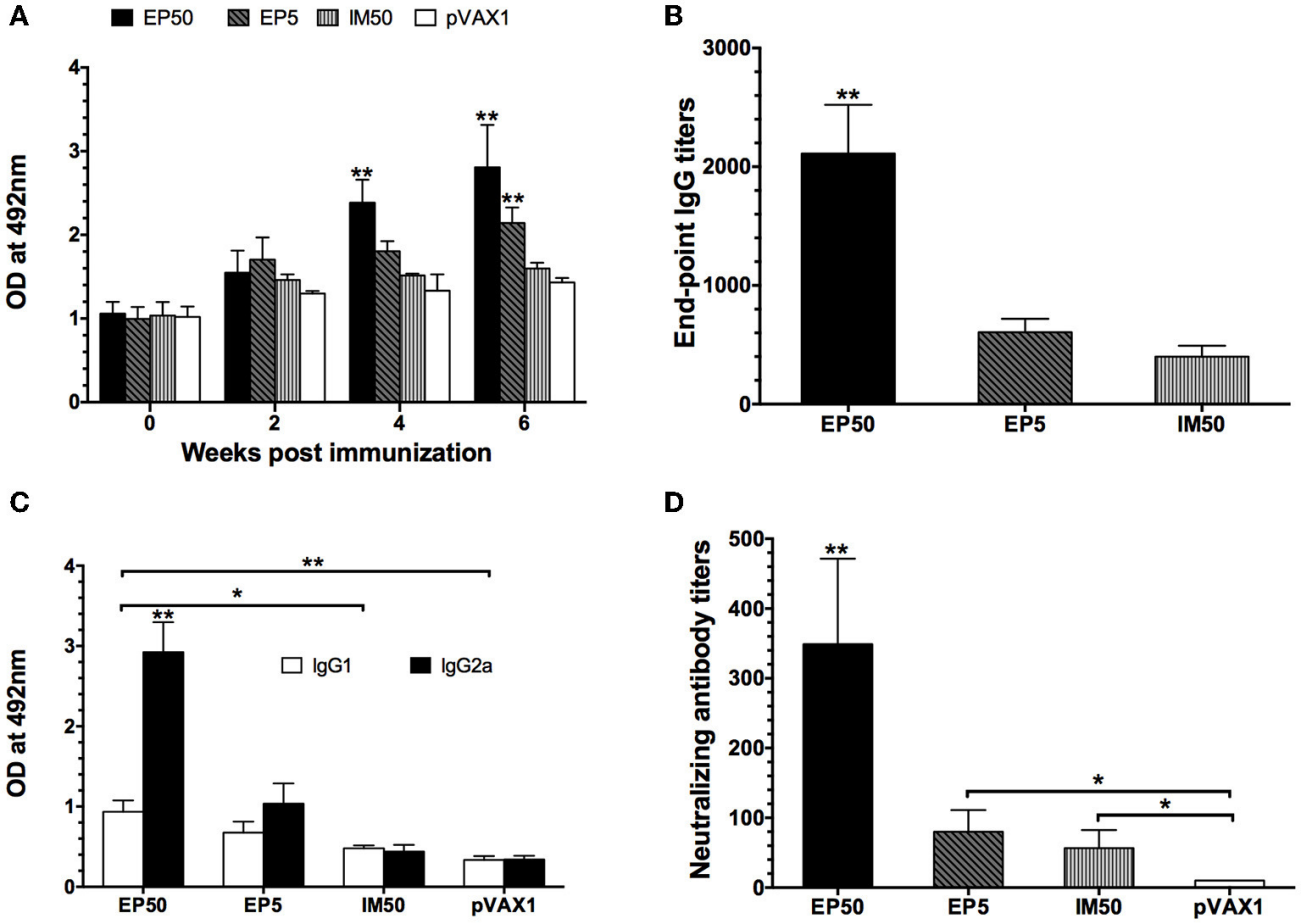
**DV1-specific antibody responses in mice sera. (A)** Dynamics of the IgG responses detected by ELISA. The mice were immunized three times at two-week intervals. Sera were collected at weeks 0, 2, 4, and 6. All samples were diluted at 1:800 (*n* = 6). **(B)** End-point titers of anti-DV1 antibodies assayed by ELISA (*n* = 6). Sera were collected two weeks after the third immunization. The results were expressed as GMT + standard deviation (SD). **(C)** The anti-DV1 IgG subclasses in mice sera determined by ELISA. Sera were collected two weeks after the third immunization. Values of IgG2a and IgG1 were reported as the mean OD + SD at 492 nm at a serum dilution of 1:100 (*n* = 8). **(D)** Serum NAb responses assayed by PRNT_50_ (*n* = 8). Sera were collected two weeks after the third immunization. NAb titers were recorded as GMT + SD. ^*^p < 0.05, ^**^p < 0.01.

The dynamic anti-DV1 IgG response at various time points was analyzed by ELISA. As shown in Figure 2A, the DV1-specific IgG level in the EP50 group showed an increased trend after the prime immunization, attained a significantly high level after the second immunization, and then maintained the highest level during the observed period. In the EP5 group, increased anti-DV1 IgG levels were observed, but the amplitude was limited compared with that in the EP50 group. However, the antibody response in the IM50 group showed no obvious change. After the third vaccination, the end-point geometric mean titer (GMT) of the IgG antibody in the EP50 group reached 1:2111 (Figure 2B), which was significantly different from that in the EP5 (1:606, *p* < 0.01) or the IM50 (1:400, *p* < 0.01) group. These results indicated that 50 μg pVAX1-D1ME delivered by EP could induce humoral immune response more effectively.

The levels of DV1-specific IgG subclasses were determined by ELISA. In general, the IgG1 and IgG2a isotypes are associated with the Th2 and Th1 immune responses, respectively. As shown in Figure 2C, the EP50 and EP5 groups elicited higher levels of IgG2a than IgG1 with mean IgG2a/IgG1 ratios of 3.90 and 3.39, respectively. The IM50 group induced almost equal levels of IgG1 and IgG2a, which was similar to the control group. IgG2a levels in mice inoculated via EP were significantly higher than those in the control and IM groups (*p* < 0.01), indicating that immunization with the pVAX1-D1ME via EP induced a bias toward the Th1 immune response, whereas vaccination via IM showed no obvious bias.

Two weeks after the third immunization, serum samples were collected to detect the anti-DV1 NAb level by PRNT_50_. As shown in Figure 2D, the highest NAb level was observed in the EP50 group with a GMT of 1:349, while NAb levels in the EP5 and IM50 were 1:80 and 1:57, respectively. There were significant differences in NAb titers between the experimental groups (EP50, EP5 and IM) and the pVAX1 group (*p* < 0.01 or *p* < 0.05).

### Cytokine generation to immunization with pVAX1-D1ME

**Figure 3:**
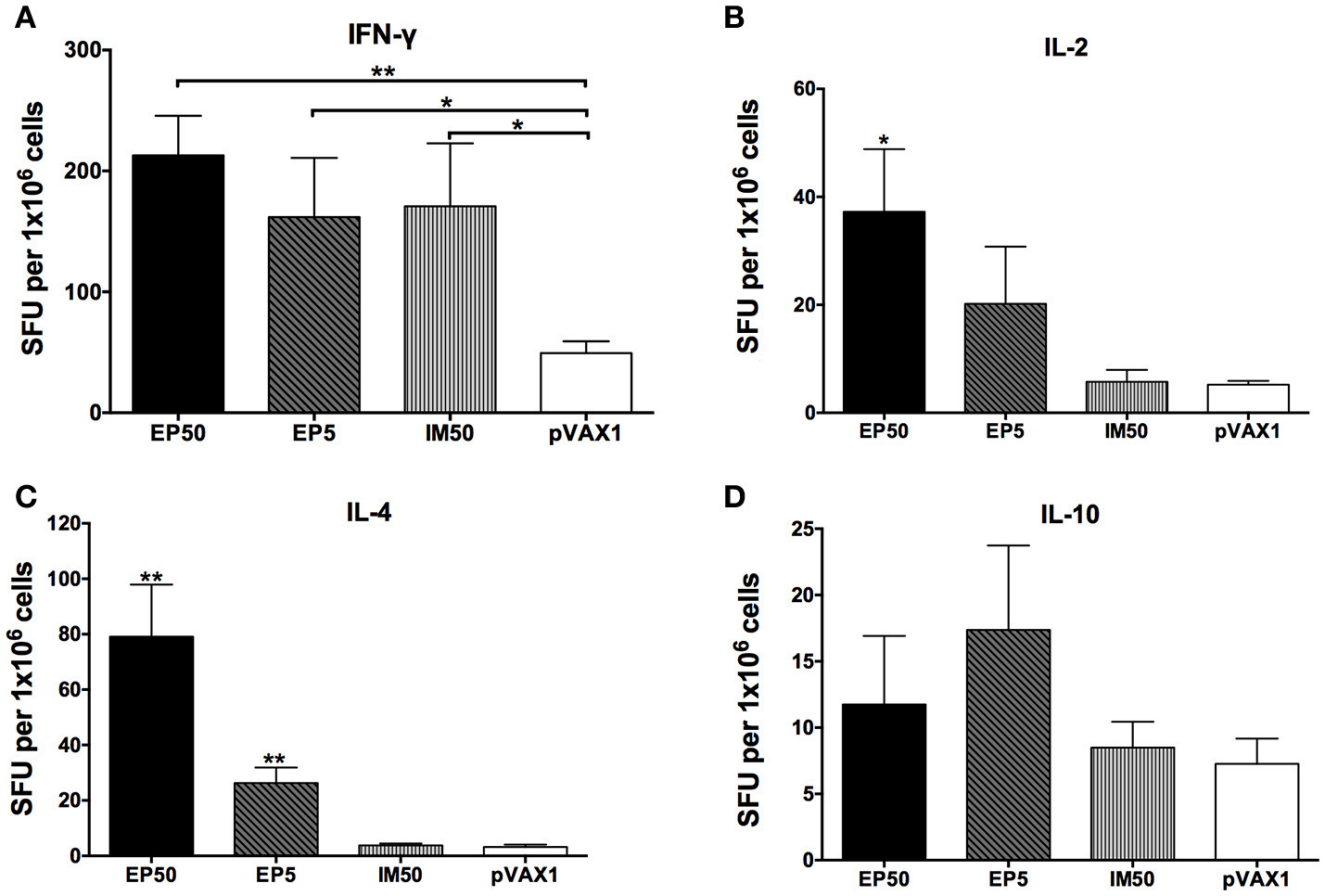
**The levels of splenocyte-secreted IFN-γ (A)**, IL-2 **(B)**, IL-4 **(C)**, and IL-10 **(D)** cytokines by ELISPOT assays (*n* = 6). Splenocytes were isolated two weeks after the third immunization. The numbers of cytokine-positive cells were recorded as the mean SFU/1 × 10^6^ splenocytes + SD. ^*^p < 0.05; ^**^p < 0.01.

Cytokines secreted by splenocytes upon stimulation with the DV1 antigen were detected by ELISPOT assay. As shown in Figure 3, levels of IFN-γ, IL-2, and IL-4 increased markedly in the EP50 group (*p* < 0.01 or *p* < 0.05) compared to the pVAX1 group. In the EP5 group, levels of IFN-γ and IL-4 increased significantly (*p* < 0.01 or *p* < 0.05) compared to the pVAX1 group. In the IM50 group, only the increased IFN-γ level was observed (*p* < 0.05). There was no significant change in IL-10 levels in all groups. Generally, IFN-γ and IL-2 are defined as markers of the Th1 response and IL-4 is defined as a marker of the Th2 response; the above results suggested that both the Th1- and Th2-type immune responses were effectively evoked by 50 μg pVAX1-D1ME via EP administration.

### Cell-mediated immune response to immunization with pVAX1-D1ME

**Figure 4:**
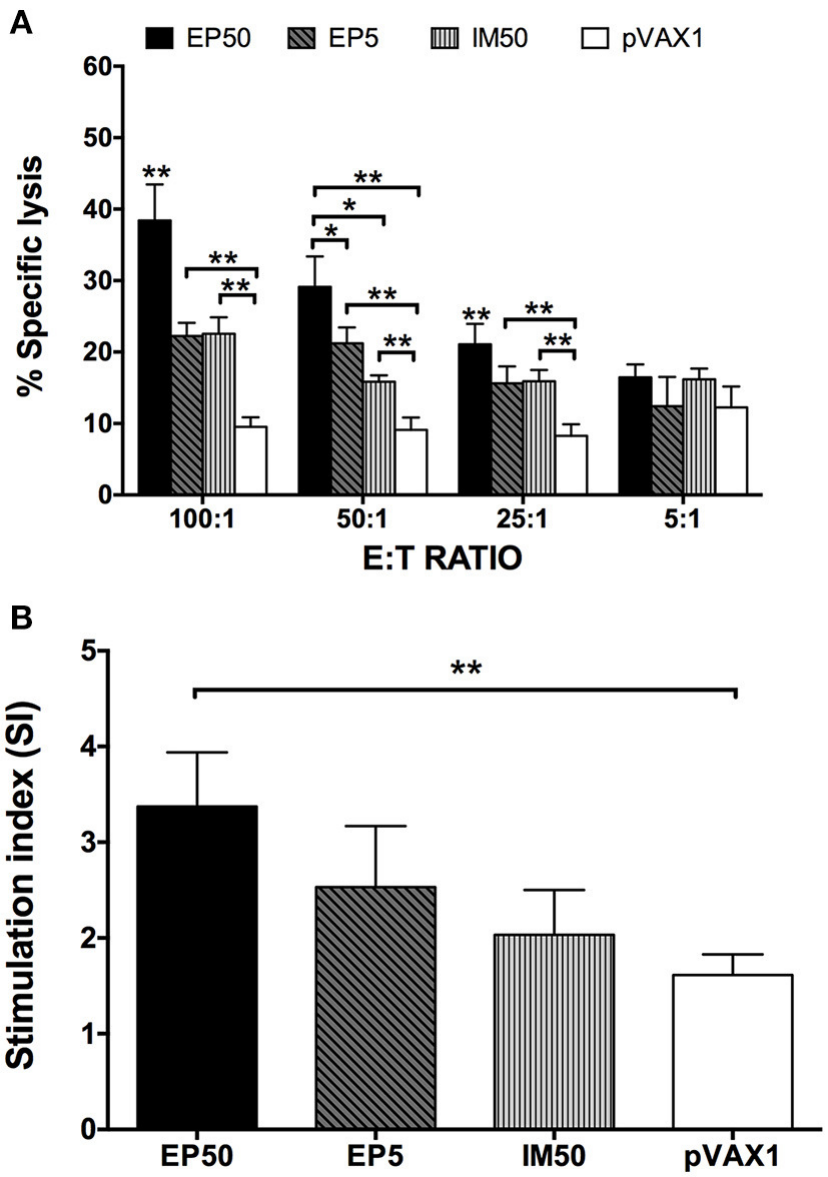
**DV1-specific cell-mediated immune responses in DNA-immunized mice**. Splenocytes were isolated two weeks after the third immunization. **(A)** DV1-specific CTL response (*n* = 6). Percentages of specific lysis + SD were shown at different E:T ratios. **(B)** Lymphocyte proliferation measured with CCK-8 (*n* = 6). The results were recorded as the mean SI + SD. ^*^p < 0.05, ^**^p < 0.01.

Splenocytes isolated from immunized mice were stimulated with the DV1 antigen, and the specific CTL activity was determined. As shown in Figure 4A, increased CTL activity was observed in both the EP and IM groups, with an E:T ratio-dependent pattern, but not in the control group. The EP50 group showed significantly increased CTL activity based on E:T ratios ≥ 25:1 and then maintained the highest CTL activity among all groups. When the E:T ratio was 100:1, the CTL activity was 38.4% in the EP50 group, with a significant difference (*p* < 0.01) from the activities in the EP5 group (22.2%) and the IM50 group (22.6%).

The splenocytes stimulated with DV1 antigen were also used to examine lymphocyte proliferation. As shown in Figure 4B, no distinct lymphocyte proliferation was observed in the pVAX1 group (SI = 1.61 ± 0.22). However, SIs in the EP50, EP5, and IM50 groups showed an increased trend, with values at 3.37 ± 0.57, 2.53 ± 0.64, and 2.03 ± 0.47, respectively, when 100 μg/ml of DV1 antigen was used. Only the lymphocyte proliferation in the EP50 group showed a significant difference compared with that in the pVAX1 group (*p* < 0.01). Taken together, the above results indicated that immunization with pVAX1-D1ME induced not only humoral but also cellular immune responses.

### Protective immunity elicited by pVAX1-D1ME

**Figure 5:**
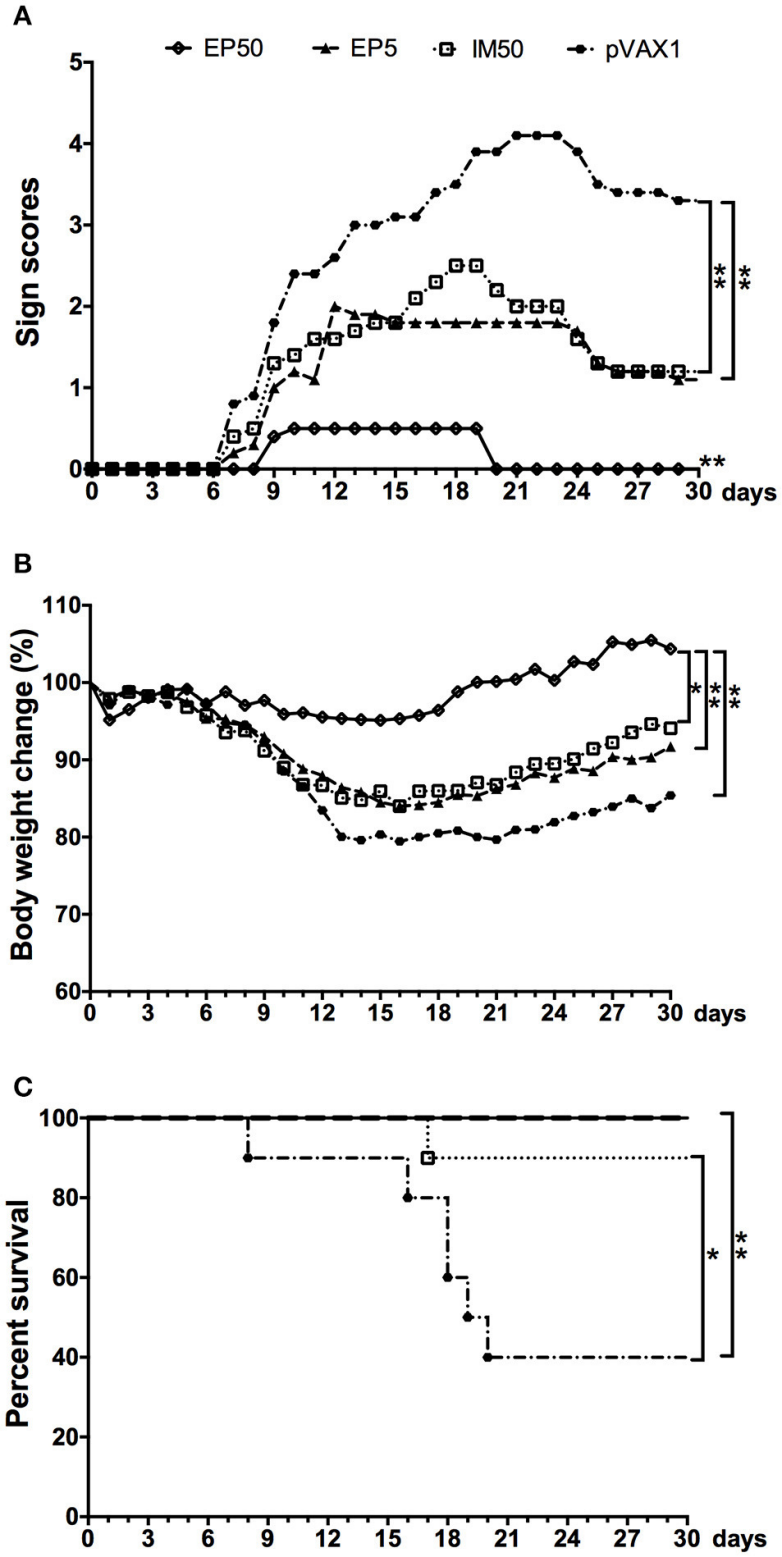
**Protective immunity elicited by the pVAX1-D1ME against DV1 infection (***n*** = 10). (A)** Pathological symptoms recorded as the mean sign scores. **(B)** The body weight reported as percentages compared to day 0. **(C)** The survival rate shown as the percentage of survivors. ^*^p < 0.05, ^**^p < 0.01.

Two weeks after the third immunization, the mice were challenged with lethal doses of DV1, and the protective efficacy of the pVAX1-D1ME DNA vaccine was evaluated. All mice in the control group showed illness at day 7 post infection, and gradually became worse (Figure 5A) with obvious body weight loss (Figure 5B). At day 16, body weight in the control group decreased by more than 21%. Finally, a total of 60% of control mice died (Figure 5C), and some surviving mice showed sequelae such as paralysis of hind limbs. In comparison, the EP50 group showed a transient and mild illness at day 9, and completely recovered without obvious changes of body weight and any sequelae during the 30-day observation period (Figures 5A,B). All mice in the EP50 group survived the viral challenge (Figure 5C). Mice in the EP5 and IM50 groups also showed illness at day 7, and clinical symptoms were more serious than those in the EP 50 group but milder than those in the control group (Figure 5A). Survival rates of 100% and 90% were seen in both the EP5 and IM50 groups with the same body weight loss of 16% (Figures 5B,C). The survival rate in all pVAX1-D1ME-vaccinated groups was remarkably different from that in the control group (*p* < 0.01, Figure 5C). The results suggested that the immunization with 50 μg pVAX1-D1ME via EP could induce significantly effective protection against the DV1 challenge with a 100% survival rate and only mild clinical symptoms.

### Determination of optimum immunization times of pVAX1-D1ME

**Figure 6:**
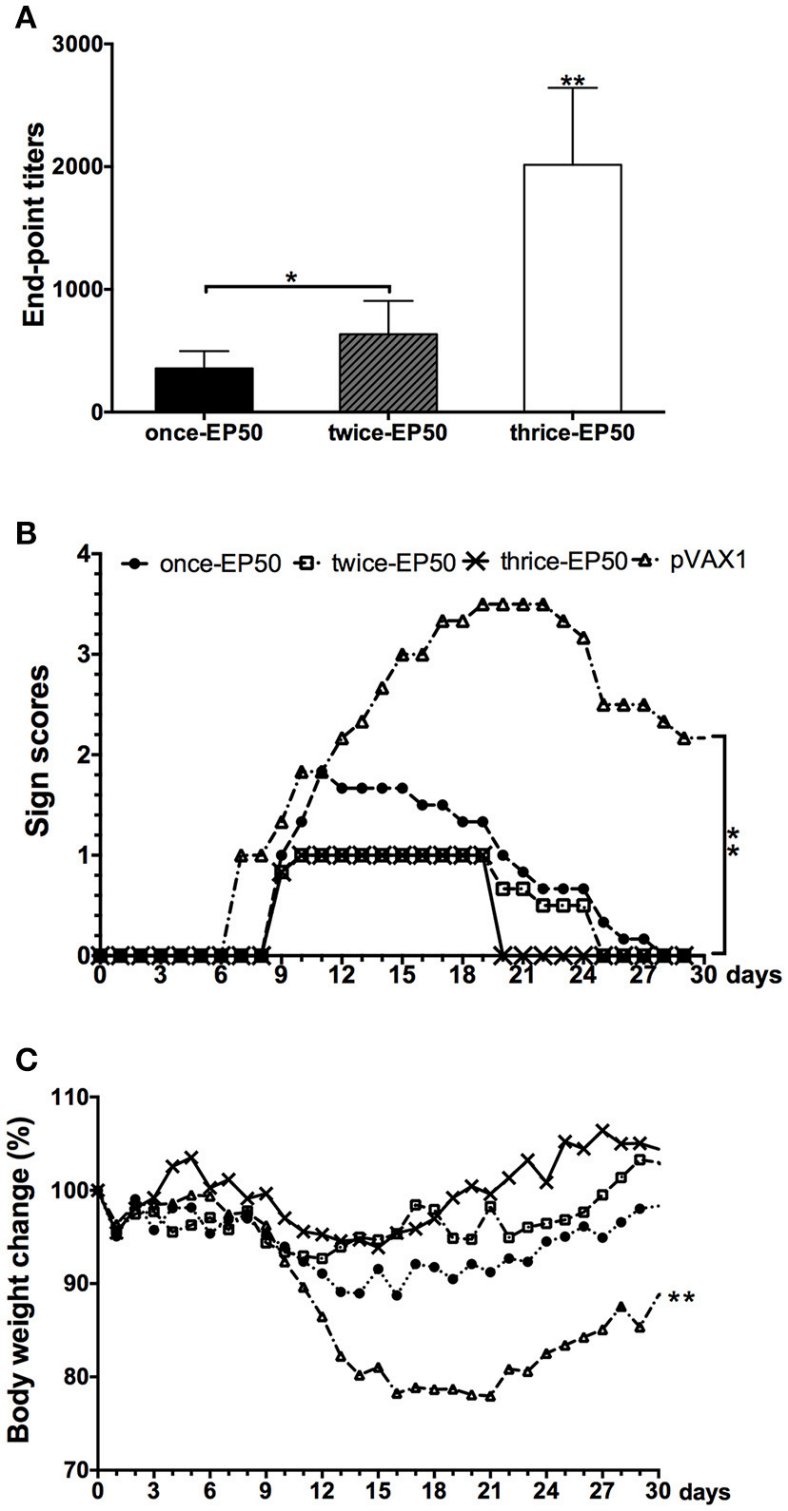
**End-point titers of anti-DV1 antibodies and protect immunity elicited by the pVAX1-D1ME under different times of vaccination (***n*** = 6). (A)** End-point titers of anti-DV1 antibodies. **(B)** Pathological symptoms recorded as the mean sign scores. **(C)** Body weight calculated as percentages compared with day 0. ^*^p < 0.05, ^**^p < 0.01.

The above results indicated that 50 μg pVAX1-D1ME via EP could induce effective immune responses and protection after immunization with three doses. To further evaluate the cost-effectiveness of vaccination, mice were immunized with 50 μg pVAX1-D1ME via EP once, twice, and three times. As shown in Figure 6A, the end-point GMT of anti-DV1 IgG was 1:2,016 in the thrice-EP50 group, which was the highest compared to that in the once-EP50 group (1:356, *p* < 0.01) and twice-EP50 group (1:635, *p* < 0.01).

After challenge with a lethal dose of DV1, the most serious symptom was observed in the pVAX1 group, but relatively mild clinical signs were observed in three pVAX1-D1ME-vaccinated groups (Figure 6B). In the pVAX1 group, mice showed illness beginning at day 7, some mice still showed sequelae within 30 days (Figure 6B), and the most notable body weight loss was 22% (Figure 6C), which was the highest among the four groups (*p* < 0.01). Meanwhile, all the vaccinated mice started to show illness at day 9, followed by differential duration of disease with various body weight losses. The symptoms of the mice in the thrice-EP50 group were the mildest; the mice completely recovered within 21 days (Figure 6B), and the most notable body weight loss was only 6.1% (Figure 6C). The mice in the once-EP50 group and twice-EP50 group gradually recovered within 28 days and 26 days (Figure 6B), and the most marked body weight losses were 11.3 and 7.3%, respectively (Figure 6C).

The aforementioned results demonstrated that three doses of pVAX1-D1ME via EP significantly ameliorated the immune response and protection and were necessary for vaccination.

### Humoral immune response and protection induced by the bivalent vaccine

**Figure 7:**
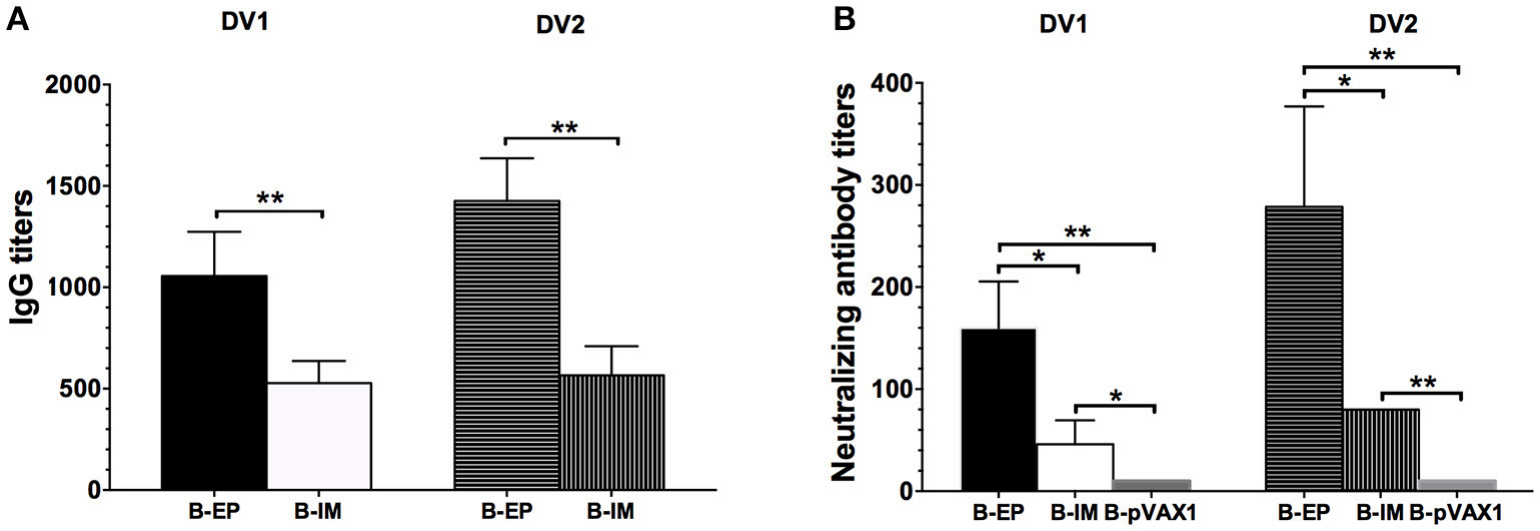
**DV1- and DV2-specific antibody responses in mice immunized with the bivalent vaccine (***n*** = 5)**. Sera were collected two weeks after the third immunization. **(A)** End-point titers of anti-DV1 and anti-DV2 IgG assayed by ELISA. The results were recorded as GMT + SD. **(B)** End-point titers of anti-DV1 and anti-DV2 NAb assayed by PRNT_50_. The results were recorded as GMT + SD. ^*^p < 0.05, ^**^p < 0.01.

To investigate the humoral immune response elicited by the bivalent vaccine, serum samples from the immunized mice were collected to analyze the specific IgG titers and NAb titers. After the final vaccination, both the DV1-specific and DV2-specific IgG levels (Figure 7A) and neutralizing activity (Figure 7B) in the B-EP group were significantly higher than those in the B-IM group. In the B-EP group, the end-point GMTs of anti-DV1 and anti-DV2 IgG were 1:1,056 and 1:1,425, respectively, which were almost two times higher than those in the B-IM group. Meanwhile, the GMTs of anti-DV1 and anti-DV2 NAbs in the B-EP group were 1:160 and 1:279, respectively, which were 3-fold higher than those obtained from the mice immunized via IM. The DV1-specific and DV2-specific IgG levels as well as the neutralizing activities within B-EP group did not show significant differences (*p* > 0.05), indicating a balanced immune response to DV1 and DV2 induced by the bivalent vaccine.

**Figure 8:**
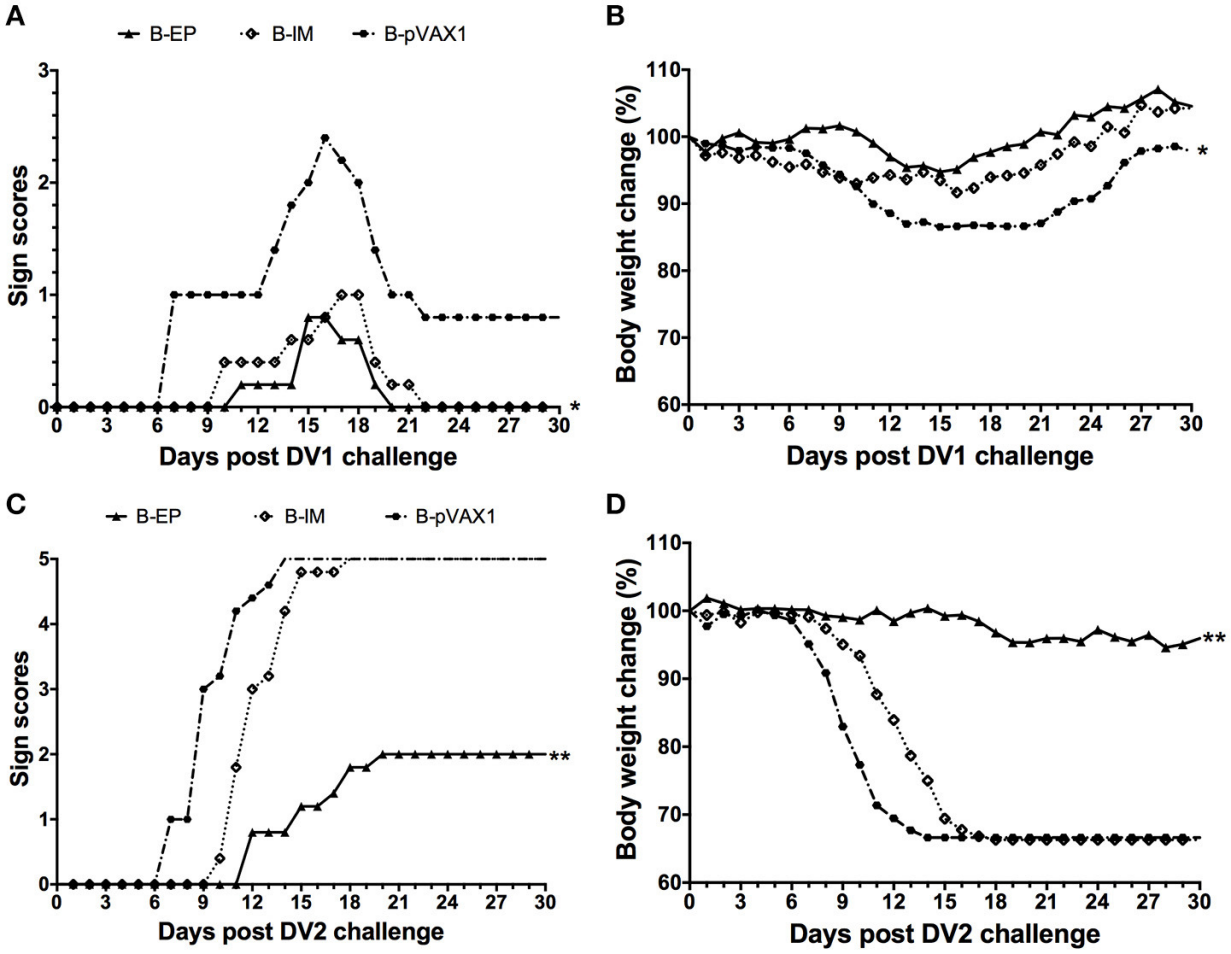
**Protective immunity elicited by the bivalent DNA vaccine against DV1 and DV2 infection (***n*** = 5). (A,C)** Pathological symptoms recorded as the mean sign scores. **(B,D)** The body weight reported as percentages compared to day 0. ^*^p < 0.05, ^**^p < 0.01.

To evaluate the protective efficacy of the bivalent DNA vaccine, two weeks after the third vaccination, mice were challenged with a lethal dose of DV1 or DV2. After challenge with DV1, the most severe symptom was observed in the B-pVAX1 group, which started at day 7 and became progressively worse (Figure 8A); the maximum body weight loss was 13.5% (Figure 8B). In the B-IM group, the symptoms started at day 10, and the mice completely recovered at day 22, with a maximum body weight loss of 8.4%. The mildest symptom was observed in the B-EP group, which began at day 11; all mice recovered absolutely at day 20 with only 5.3% maximum body weight loss. Similarly, after challenge with DV2, the B-pVAX1 group showed the most severe symptom, which began at day 7 and then became rapidly worse; all mice died till day 14 with a 33.4% body weight loss (Figures 8C,D). The mice in the B-IM group became sick at day 10; all mice died at day 18 with a 33.7% body weight loss. Meanwhile, the B-EP group showed the mildest symptom, which began at day 12; all mice survived, and the maximum body weight loss was only 5.4%. Importantly, there were no increased clinical signs observed in mice immunized with the bivalent vaccine after either DV1 or DV2 challenge, although there is theoretical risk of immune enhancement.

## Discussion

During the last 50 years, several groups have made concerted efforts in developing dengue vaccines, and significant progress has been made (Thomas and Endy, 2011; Guzman and Harris, 2015). The recombinant, live, attenuated, chimeric yellow-dengue tetravalent dengue vaccine (CYD-TDV, Dengvaxia) produced by Sanofi Pasteur is the most promising (Guy et al., 2015). CYD-TDV was structured using the backbone of the yellow fever 17D vaccine strain by replacing the *prM* and *E* genes with those of DV1-4 (Flipse and Smit, 2015). The most noticeable benefit of the vaccine was the large reduction in hospitalization of dengue by 67% to 80%, but vaccination-related side effects occur in children younger than 9 years old (Capeding et al., 2014; Da Costa et al., 2014; Dorigatti et al., 2015; Villar et al., 2015). Although, the CYD-TDV has been recently registered in several countries, the United States still vigorously promoted the research of phase 3 clinical trials of the attenuated dengue vaccine developed by the NIH in Brazil (NIAID News Releases, 2016). Therefore, it is necessary to develop safer, more economical and effective DV vaccines.

DNA vaccines have been developed for decades with a number of potential advantages as mentioned above (Khan, 2013). However, as a novel methodology in the prevention of infectious diseases, DNA vaccination has not yet achieved much success in large animals, mainly due to the insufficient immunogenicity, and no licensed DNA vaccine is currently available in humans. Several technical improvements have been investigated, and EP is considered as one of the most promising delivery strategies and has been used in many non-human research and human clinical trials (Lu et al., 2008; Sardesai and Weiner, 2011). In Australia, a growth hormone-releasing hormone plasmid delivered by EP has been approved in the treatment of pigs (Person et al., 2008). In our recent studies, the DNA vaccine candidates inoculated using EP showed enhanced expression of the antigen in local sites, strong immune response and protective efficacy in mice, rabbits and pigs (Chen et al., 2016; Sheng et al., 2016). Thus, in the present study, we constructed the DNA vaccine candidate pVAX1-D1ME and explored *in vivo* EP for gene delivery in comparison with traditional IM injection in mice. Immunization with three doses of 50 μg pVAX1-D1ME via EP not only elicited a consistently higher IgG response and higher NAb titer but also generated higher specific CTL activity, a stronger lymphocyte proliferative response and higher levels of splenocyte-secreted IFN-γ, IL-2, and IL-4. Our results indicated that three doses of 50 μg pVAX1-D1ME via EP could induce both antibody- and cell-mediated immune responses in mice.

Although, there was no significant difference in survival rates among the EP50, EP5, and IM50 groups in the protection test (Figure 5), the severity of clinical signs was different. The EP50 group only had transient mild illness with unchanged body weight, while the EP5 and IM50 groups showed moderate illness with 16% of body weight loss. This result was much better than that observed in our previous study (Zheng et al., 2011). In the latter, although the same survival rate could be achieved by 100 μg of the DNA pCAG/DV1/E (expressing the prM and E proteins of DV1) inoculated by IM without EP, immunized mice showed obvious illness. EP delivery only requires one-tenth of the DNA dose used in IM injection but enhances plasmid delivery by 100–1,000-fold; gene expression then induces a more effective immune response (Wang et al., 2014). Therefore, in combination with humoral and cellular responses, the results suggests that *in vivo* EP with a suitable plasmid dose is a promising strategy for DV DNA vaccination, and three immunizations at 50 μg pVAX1-D1ME are necessary for inducing effective immune response and protection. A recent report by Prompetchara et al. demonstrated that a tetravalent prME DNA vaccine candidate administered by EP could induce high levels of NAb against all DV serotypes (Prompetchara et al., 2014), which supports our results and suggests that EP is an important method for further developing a tetravalent vaccine.

Ideal multivalent dengue vaccines should induce a balanced immune response to all DV serotypes. In this study, the immune response and protection induced by the bivalent vaccine against DV1 and DV2 were preliminarily evaluated in mice. We found that immunization via EP could induce similar levels of anti-DV1 and anti-DV2 IgG antibodies as well as NAb titers, indicating balanced humoral immune responses to two serotypes. Notably, immunization with the bivalent vaccine could reduce illness severity and body weight loss, indicating a protective effect. However, the results against challenge with DV1 showed some differences from cases of challenge with DV2. The B-EP and B-IM groups showed similar results in reducing severity of illness and limiting body weight loss against DV1. After DV2 challenge, only the B-EP group exhibited protection compared with the B-IM and control groups. This result was consistent with our recent study, in which the pVAX1-D2ME via IM failed to provide full protection (Chen et al., 2016). The symptoms were not aggravated in the mice immunized with the bivalent vaccine via EP after challenge with DV1 or DV2, and 33% of body weight loss after DV2 challenge was similar with those in the monovalent DV2 vaccine-immunized mice (Chen et al., 2016), meaning no ADE occurred. In combination with the results in our previous study, the effectiveness of the pVAX1-D1ME vaccine candidate is mainly attributed to the suitable immunogen and EP delivery system. Meanwhile, it was noted that the end-point titers of anti-DV1 and anti-DV2 in the bivalent vaccine-immunized mice were lower than those in the monovalent vaccine-immunized mice, indicating interference between the DV1 and DV2 vaccine candidates. This evidence should be considered in further research on DV tetravalent vaccine. It has been reported that dose adjustment, multisite vaccination, and immunologic priming against DV and other flaviviruses might reduce interference in DV vaccines (Anderson et al., 2011).

In summary, this study demonstrated that administration with three doses of 50 μg pVAX1-D1ME via EP induced persistent humoral and cellular immune responses and effectively protected mice against lethal DV1 challenge. Immunization with the bivalent vaccine via EP generated balanced antibody responses and protection against DV1 and DV2 without increased clinical signs. Our results suggest a promising method for developing a DV tetravalent DNA vaccine.

## Author contributions

XZ: Performed research, analyzed data, and wrote paper. HC: Designed research, analyzed data, and revised paper. RW: Helped with experiment and analyzed data. DF, KF and NG: Helped with experiment. JA: Designed research and revised paper. All authors have read the manuscript and approved its submission. XZ and HC have equal contribution to this work (Co-first authors).

## Funding

We acknowledge the financial support by the National Natural Science Foundation of China (81372935, 81271839, 81671971, 81471957, and U1602223).

### Conflict of interest statement

The authors declare that the research was conducted in the absence of any commercial or financial relationships that could be construed as a potential conflict of interest.
